# Impact of Scene Features of E-Commerce Live Streaming on Consumers’ Flow and Purchase Intentions of Sporting Goods

**DOI:** 10.3390/bs15020238

**Published:** 2025-02-19

**Authors:** Zhengyu Li, Yan Wang, Beth Anne Cianfrone, Zhen Guo, Bo Liu, James Zhang, Chenyu Shi

**Affiliations:** 1 Division of Sports Science and Physical Education, Tsinghua University, Beijing 100084, China; lizhengy23@mails.tsinghua.edu.cn (Z.L.);; 2 School of Economics and Management, Shanghai University of Sport, Shanghai 200438, China; 3 Department of Kinesiology and Health, Georgia State University, Atlanta, GA 30303, USA; 4 Department of Kinesiology, University of Georgia, Athens, GA 30602, USA

**Keywords:** online shopping, e-commerce programming, sport merchandising, immersive experience

## Abstract

Purpose—The purpose of this study is to examine the mechanism of scene features (i.e., visual appeal, presence, and scene–product matching) of e-commerce live streaming and how it impacts consumers’ flow and purchase intentions of sporting goods. Design/methodology/approach—To test the hypotheses in the conceptualized model that was developed based on the Stimulus–Organism–Response (S-O-R) model, sports consumers (*N* = 340) who watched e-commerce live streaming responded to an online survey administration. Structural equation modeling (SEM) was applied to examine the research model and test the hypotheses. Findings—The results reveal that scene features positively affect consumer flow experience, in which scene–product matching is the main contributing factor. Moreover, the flow experience plays a critical mediating role in influencing purchase intentions; meanwhile, the moderation role of sport identification was not found to be statistically significant. Originality/value—This study fills the void to explore how scene features of e-commerce live streaming influence consumer behavior associated with sporting goods merchandise through immersed flow experience. Confirming the applicability of the S-O-R model in the sports e-commerce live streaming setting, the findings of this study identify dimensions of scene features of e-commerce live streaming and highlight the significance of developing scene–product congruence features when designing, operating, and promoting live streaming programs while enhancing immersive involvement.

## 1. Introduction

With the rapid advancement of digital technology, live streaming has become a growing trend. For example, the number of live streaming users in China reached 765 million, comprising 71% of total internet users. Among them, e-commerce live streaming users reached 526 million, representing 48.8% of the total internet population. In 2022, the transaction volume for e-commerce live streaming reached CNY 3.5 trillion (USD 0.48 trillion) (China Internet Network Information Center, 2023). E-commerce live streaming began when Taobao integrated live streaming functionality in 2016. Following this transformation, e-commerce platforms and short video apps have been heavily invested in traffic, capital, and human resources to cultivate this business sector. The year 2019 marked a significant milestone, often referred as the first year of China’s e-commerce live streaming, which was evidenced by the fact that major platforms like Taobao, Douyin (TikTok China), and Kuaishou experienced explosive growth in transaction volumes. E-commerce, such as Amazon (leading online retailer with an app) and TikTok (social media platform) in the United States and Taobao (leading online retailer with an app), Douyin (social media platform), and Kuaishou (social media platform) in China, is driving the trend of online shopping. Short video platforms, like TikTok, Douyin (TikTok China), and Kuaishou, may have influencers or advertisers discussing products for sale. As such, the way in which these products are presented to the audience visually, including the background and the scene features, is an important part of the influence process of sales. Existing studies have mostly focused on single countries, such as Indonesia (Chandraa et al., 2024), Malaysia (W. X. Hong & Hoo, 2022), and Thailand (Chandrruangphen et al., 2022). Ni and Ueichi (2024) conducted a comparative study of e-commerce live streaming in the United States, China, and Japan and found that consumers’ perceptions and behaviors vary due to different cultural backgrounds. In the field of e-commerce live streaming, China has developed very rapidly and holds significant research value.

The sporting goods industry in China, valued at CNY 1.9 trillion (USD 26 billion), constitutes 59.6% of the nation’s sports industry GDP (National Bureau of Statistics, 2023). The COVID-19 pandemic severely disrupted sports events and the sporting goods supply chain, resulting in decreased foot traffic and sales in brick-and-mortar stores, leading many well-known brands to close their physical locations (Reuters, 2020). Paradoxically, the pandemic also heightened global awareness of health and fitness, driving a trend toward home-based workouts and presenting new challenges and opportunities for the sporting goods market. Live streaming emerged as a valuable tool for retailers to increase sales during this period (S. Zheng et al., 2023). Consequently, many sporting goods brands began leveraging live streaming platforms to explore innovative consumer involvement and engagement strategies and boost sales. Relevant studies on specific product types have predominantly focused on experience products (e.g., clothing and cosmetics (Lu & Chen, 2021), fresh produce (Yang et al., 2021), and travel products (X. Liu et al., 2022)). Previous research findings also revealed that the same stimuli might have varying effects on different product categories (Park & Lin, 2020; Xu et al., 2020). Sporting goods possess unique characteristics—such as specific usage environments, product technology, and user experience—that warrant more targeted and concerted research efforts to avoid the ineffective application of findings derived in other industries.

This study applies a conceptual framework built on the Stimulus–Organism–Response paradigm (Mehrabian & Russell, 1974) and the flow theory (Csikszentmihalyi, 1975) to analyze whether scene features in live streaming can enhance consumers’ flow experience, thereby influencing their purchase intention in the context of sporting goods e-commerce. The study contributes in three significant ways. First, its specificities are the dimensions of distinct scene features of sporting goods e-commerce live streaming: visual appeal, presence, and scene–product matching. Second, following the conceptual framework, it examines how these scene features impact purchase intentions in the sporting goods setting. Third, it uses scene features as independent variables, flow experience as a mediating variable, and sport identification as a moderating variable, exploring the mechanisms by which these concepts interact to influence consumer purchase intentions. This research offers a meaningful extension to the literature on e-commerce live streaming and the sports industry.

## 2. Literature Review

### 2.1. Stimulus–Organism–Response Theory (S-O-R Theory)

The Stimulus–Organism–Response (S-O-R) model was first proposed by Mehrabian and Russell (1974), which holds that cue (stimulus, S) perceived by an individual from the environment can influence a person within the cognitive or emotional state (organism, O), resulting in positive or negative behavior in response to the stimulus (response, R). The theory is widely used in research fields such as environmental psychology and consumer behavior.

Donovan and Rossiter (1982) first applied the S-O-R model to the field of consumer behavior, deeming that the store environment can stimulate consumers’ perception and then affect consumers’ behavior. Eroglu et al. (2001) applied the theory to the online retailing context for the first time and built (and later confirmed (Eroglu et al., 2003)) a model of the impact of the online shopping environment on consumers’ purchase intention. In traditional e-commerce studies, scholars have explained the influence of stimuli on consumers’ intention and behavior through website characteristics, design, background color, and online comments (Y. F. Chen & Wu, 2016; Ettis, 2017). Along with e-commerce entering into the era of live streaming, a large number of studies have emerged to explain consumer behavior in this field by using the S-O-R model. Fei et al. (2021) used an eye-tracking experiment to study the influence of social cues (i.e., herding message, interaction text) on consumers’ attention allocation process and purchase intention. Ming et al. (2021) confirmed that social presence (i.e., live streaming platform, viewers, and streamers) would influence consumers’ impulsive buying behavior through trust and flow state as mediators.

In sporting goods e-commerce live streaming, consumers would also be stimulated by the scene to a certain extent, which induces the organism to change cognition or emotion, further affecting the purchase intention and behavior. Therefore, this study explores the factors affecting consumers’ purchase intention based on the S-O-R model, in which scene features are used as external stimuli (S), the flow experience as consumers’ internal organismic response factors (O), and consumers’ purchase intention is used as the resulted response (R).

### 2.2. Scene Features of Sporting Goods E-Commerce Live Streaming

Although e-commerce live streaming has received plenty of attention in practice, there is a paucity of understanding about it in the research area (Gu et al., 2023). Prior research had found a series of factors that could affect consumers’ purchase behaviors in e-commerce live streaming. It is mainly divided into three perspectives, platforms (e.g., usefulness, ease to use, convenience, IT affordance; Hyun et al., 2022; S. C. Lin et al., 2022; Sun et al., 2019), anchors (e.g., attractiveness, interactivity, expertise; Hou et al., 2019; F. Liu et al., 2020; Rungruangjit, 2022; S. Zheng et al., 2023), and consumers (e.g., perceived value, perceived uncertainty, self-product fit; J. Guo et al., 2021; Park & Lin, 2020). An e-commerce live streaming consists of three essential elements—people, goods, and scenes, which refer to the anchor, product, and live scenes, respectively. Scoble and Israel (2014) believed that the scene would subvert the social service industry, and the scene design would gradually become an important basis for business model innovation and reform. Scene is the key element of retailing and the core of e-commerce live streaming, with four values: emotional, marketing, social, and communication. It can be divided into tangible elements (e.g., equipment, spatial layout, goods display) and intangible elements (e.g., music, lighting, technology applications). However, most studies only incorporate it into the overall characteristics of e-commerce live streaming rather than separate studies like the characteristics of anchors. Moreover, the underlying mechanism by which scene features influence consumers’ purchase intention is still unknown.

This study chooses Visual Appeal (VA), Presence (PR), and Scene–Product Matching (SM) as the scene features of sporting goods e-commerce live streaming. This focus was based on the consideration that the designed features of business-to-customer provisions are essential to ensure meeting the operational quality standards when staging live streaming. While there are merits with this focus, not including interactive elements, such as user engagement and comments, can overlook valuable information from the consumer perspective. In this study, we focused more on the scene features that sports vendors can actively and significantly adjust, particularly the visual aspects that consumers can directly see. However, we recognize the importance of interactive elements in influencing consumers’ purchase intentions. In traditional e-commerce settings, interactivity primarily revolves around consumer–machine interactions, but the main emphasis of interactivity has shifted to the interactions between anchors and customers or among customers in the e-commerce live streaming context, owing to technological advances in mobile commerce (Xue et al., 2020). In live streaming commerce, a consumer can interact with an anchor and other consumers, and this interaction takes place in real time. We considered it as one of the anchor characteristics; hence, they were not included in the scene characteristics. Additionally, real-time user engagement and influencer communication are often discussed from the perspective of e-commerce live streaming platforms and technologies relating to the perceived ease of use within the Technology Acceptance Model (TAM). On platforms like Douyin (TikTok China), consumers can participate in comments and interactions. Thus, we argue that it is difficult for sports vendors to fully control these elements.

VA refers to visual accessibility, and a good visual experience will have a positive impact on consumers’ emotions and perceptions. Tong et al. (2022) indicated that the screen size of consumers watching e-commerce live streaming is limited, and the background of live streaming may affect consumers’ emotions and responses. Y. Liu et al. (2013) found that the visual appeal of a website can cause consumers to make an online impulse purchase. Therefore, the scene of e-commerce live streaming should allow consumers to have a more intuitive visual experience of the product, and it is necessary to design a scene that is visually attractive to customers.

The concept of PR originated in the field of communication and was divided into physical presence and social presence (Ijsselsteijn et al., 2000), both of which can enhance consumers’ online purchase intention (Nichaya & Joseph, 2010). Physical presence describes the illusion of being physically present in the setting simulated by the medium, such as the computer-mediated environment (Khalifa & Shen, 2004). However, social presence refers to the sense of “being together with another” (e.g., human contact, human warmth, and sensitivity) (Biocca et al., 2003; Ou et al., 2014). This study mainly focused on the scene features of e-commerce live streaming, so PR primarily refers to physical presence, which emphasizes providing detailed and visible product information to customers as if they were watching the product and obtaining product information at the anchor’s location (Li, 2019).

The “match-up” theory was formally proposed by Kamins (1990), who believed that the consistency between celebrity and product determines the effect of advertisement, that is, the higher the degree of matching between the two, the better the effect of advertisement. Mitchell et al. (1995) demonstrated the impact of consistency between store design (e.g., background, lighting, and music) and products on the customer purchase experience. The SM will build a vivid shopping environment and attract consumers to browse for a longer time. Moreover, the presentation of the product appears more prepared and predictable (A. Y. Lee & Labroo, 2004), effectively helps consumers find the products or information they need (X. Zheng et al., 2019), and brings consumers a more authentic sense of experience and a stronger sense of identification with the products. Shang et al. (2023) confirmed that background–product matching in e-commerce live streaming can positively affect consumers’ cognition and further influence consumers’ purchase intention.

### 2.3. Flow Experience

Csikszentmihalyi (1975) first proposed the concept of Flow Experience (FE) to explain the phenomenon of when people are doing something they love, irrelevant thoughts and feelings will be filtered out, and people will focus on it without reward, enjoy it, and forget the surrounding environment and even time. Csikszentmihalyi and LeFevre (1989) further pointed out that the FE refers to the inner feeling that people obtain when they put their heart and soul into an activity. Hoffman and Novak (1996) deemed the FE as an intrinsic pleasure on a spiritual level, which is similar to the “peak experience” in Maslow’s hierarchy of needs theory and the “peak performance” proposed by Privette (1983), but there are some differences among the three. Specifically, peak experience has differences in source, degree, and span time with FE. The peak performance and flow experience differ in the degree of enjoyment; moreover, the former has a strong self-consciousness, while the latter performance has a loss of self-consciousness. FE has been extensively studied in the fields of sports, leisure, education, psychological health, and work efficiency. Hoffman and Novak (1996) first introduced FE into the internet field, considering it as a state of internet browsing. Several systematic reviews have been conducted to summarize the application of the flow concept across various domains (Jackman et al., 2019; Tan & Sin, 2021). Y. Zhang and Wang (2024) used bibliometric techniques to analyze 2622 literature related to flow experience and identified three theoretical themes (mechanism, positivity, and health) and five applied themes (technology, gaming, sports, creativity, and education) on which researchers have focused. A vast array of existing research focuses on the antecedents, experiences, and consequences of the flow experience (J. Lin et al., 2020). In the context of live-stream shopping activities, the flow theory provides a robust conceptual framework to explore how consumers make purchasing decisions within the live-stream shopping environment (Ming et al., 2021). Yin et al. (2023), by incorporating the concepts of flow theory and presence, explored how consumers enter a state of flow in live-stream shopping due to highly enjoyable experiences.

The dimensions of FE have been a concern for many scholars. From unidimensional to multi-dimensional, the characteristics of the FE dimension are shown in Table 1. However, some scholars hold a negative attitude towards the multi-dimensions of FE. Hoffman and Novak (1996) supported the single dimension and pointed out that FE is a single-dimensional variable with many antecedents and consequences. Schiefele (2013) noted that the multi-dimensional was just a different expression and description of the same attitude, which does not mean that FE has multiple dimensions. Although the dimensions of FE are debated, there are some common points. When a person experiences FE, they fully immerse themselves in the present moment, ignore the loss of time, and feel enjoyment. Moreover, many studies measured FE with concentration, enjoyment, and time distortion (transformation of time) (H. Chen, 2006; Y. M. Guo & Poole, 2009; S. M. Lee & Chen, 2010; Wu & Chang, 2005), so the measurements of FE in this study will also follow the above-mentioned common point.

## 3. Hypotheses Development

### 3.1. The Scene Features of Sporting Goods E-Commerce Live Streaming and Flow Experience

In an e-commerce live streaming, consumers’ attention changes as the physical characteristics of the stimulus change. Consumers will have more enjoyment on a visually appealing website (Y. Liu et al., 2013). Visual appeal will attract consumers to watch the live streaming and make them feel a high sense of immersion and presence (Sun et al., 2019). Therefore, a scene with a higher degree of visual appeal will make it easier for the consumer to enter into the flow experience state. Hoffman and Novak (1996) constructed a model based on a web environment indicating that presence and high concentration could induce flow experience. Subsequently, Novak et al. (2000) modified the model and demonstrated that presence and time distortion can directly lead to flow experience. J. Chen et al. (2009) also confirmed that the presence positively affected the generation of online consumers’ flow experience. Therefore, the higher the degree of presence perceived by consumers, the deeper the flow experience. Chang et al. (2010), using the example of game advertising, pointed out that placing a product in a game scene that matches it can improve the relevance of the game to the product. Scene–product matching helps consumers to integrate, process, evaluate, and memorize product information effectively (Van Rompay et al., 2010), which consumers to immerse in the e-commerce live streaming, further inducing a flow experience. Therefore, the higher the scene–product matching, the easier it is for consumers to enter into the flow experience state. Accordingly, we proposed the following hypotheses:

**H1a.**  *In sporting goods e-commerce live streaming, visual appeal positively influences consumers’ flow experience.*

**H1b.**  *In sporting goods e-commerce live streaming, presence positively influences consumers’ flow experience.*

**H1c.**  *In sporting goods e-commerce live streaming, scene–product matching positively influences consumers’ flow experience.*

### 3.2. The Scene Features of Sporting Goods E-Commerce Live Streaming and Purchase Intention

Existing research on brick-and-mortar store retail indicated that the marketing environment and visual layout of offline stores have a certain impact on consumers’ purchase intention (Khakimdjanova & Park, 2005). Similarly, in the research on online consumption, the visual appeal of a website’s page design can attract consumers to the site and positively influence purchase intention (Chi, 2018). Blanco et al. (2010) confirmed that the design of the website is a key factor for clothing vendors to achieve positive outcomes. B. Zhang et al. (2021) found that the visual appeal of e-commerce live streaming has a significant positive impact on consumers’ purchase intention. Therefore, we assume that the higher the visual appeal, the stronger consumers’ purchase intention. Online retail should create a context similar to brick-and-mortar stores to achieve the best shopping results (Lindström et al., 2016), which can be understood as presence. Therefore, we assume that the higher the presence, the stronger the consumers’ purchase intention. The more the scene of the website fits the category of the product in the advertisement, the more consumers will recognize the advertisement (Shamdasani et al., 2001). In e-commerce live streaming, scene–product matching will provide a more authentic experience and stronger product identification for consumers and then improve the purchase intention. Shang et al. (2023) first proved that the product–background fit of e-commerce live streaming can improve consumers’ perceived pleasure and further induce purchase intention. Therefore, we assumed that the higher the scene–product matching, the stronger the consumers’ purchase intention. Accordingly, we proposed the following hypotheses:

**H2a.**  *In sporting goods e-commerce live streaming, visual appeal positively influences consumers’ purchase intention.*

**H2b.**  *In sporting goods e-commerce live streaming, presence positively influences consumers’ purchase intention.*

**H2c.**  *In sporting goods e-commerce live streaming, scene–product matching positively influences consumers’ purchase intention.*

### 3.3. Flow Experience and Purchase Intention

In the context of virtual shopping, consumers can easily perceive pleasure when immersed in the experience (Yim et al., 2017), and the perceived pleasure can directly affect consumers’ purchase intention (A. Chen et al., 2017). Korzaan and Boswell (2008) believed that flow experience positively influences consumers’ purchase intention. This finding has been supported by numerous studies in the field of hotel books, travel purchases, virtual goods, game purchases (Ali, 2016; Gao & Bai, 2014; E. Huang, 2012; H. J. Liu & Shiue, 2014), and especially online shopping (mobile platform) (Gao et al., 2015; Kim & Han, 2014; Zhou, 2013). Therefore, when consumers enter into the flow experience, they will ignore time and perceive strong enjoyment, which will immerse consumers in the sporting goods e-commerce live streaming, thus generating purchase intention. Accordingly, we proposed the following hypothesis:

**H3.**  *In sporting goods e-commerce live streaming, flow experience positively influences consumers’ purchase intention.*

### 3.4. The Mediating Role of Flow Experience

The scene features (i.e., visual appeal, presence, and scene–product matching) of sporting goods e-commerce live streaming make consumers experience the feeling of shopping in the brick-and-mortar stores and concentrate on the authentic use situation of the recommended product. At this time, consumers will ignore what is happening around them and enter into the flow experience, further generating the desire to own the product. Disastra et al. (2019) found that visual appeal positively affects consumers’ purchase intention through flow experience in online shopping. Animesh et al. (2011) indicated that presence positively impacts web users’ purchase intention on virtual products through flow experience. However, there is no existing research proving that scene–product matching can influence purchase intention through flow experience in the field of e-commerce live streaming. Therefore, we assumed that the scene features would immerse consumers in the flow experience through a series of stimuli and then impact their intrinsic cognitive and affective, ultimately influencing consumers’ purchase intention. Accordingly, we proposed the following hypotheses:

**H4a.**  *In sporting goods e-commerce live streaming, flow experience mediates the effect of visual appeal and consumers’ purchase intention.*

**H4b.**  *In sporting goods e-commerce live streaming, flow experience mediates the effect of presence and consumers’ purchase intention.*

**H4c.**  *In sporting goods e-commerce live streaming, flow experience mediates the effect of scene–product matching and consumers’ purchase intention.*

### 3.5. The Moderating Role of Sport Identity

Sport identification (SI) refers to the attachment and affection of an individual to a certain sport (Wann & Branscombe, 1993). The degree of sport identification is a crucial factor affecting sports fans’ attitudes and behavior. Sports fans with a higher degree of identification will watch more games, learn more about players or teams, and be more sensitive to wins or losses. Moreover, they will purchase more products (e.g., uniforms, hats, tickets, and licensed merchandise) and related services based on the consumer behavior perspective (Kwon & Armstrong, 2002; Z. Zhang et al., 2005). Based on the context of the Social Network System (SNS), S. P. Hong and Rhee (2016) found that sport identification can moderate consumers’ purchase intention on online sporting goods. Therefore, we assumed that when the consumer watches sporting goods e-commerce live streaming and generates flow experience, the consumer with higher sport identification will perform stronger purchase intention than the lower degree. Accordingly, we proposed the following hypothesis:

**H5.**  *In sporting goods e-commerce live streaming, sport identification moderates the effect of flow experience and consumers’ purchase intention.*

**Figure 1:**
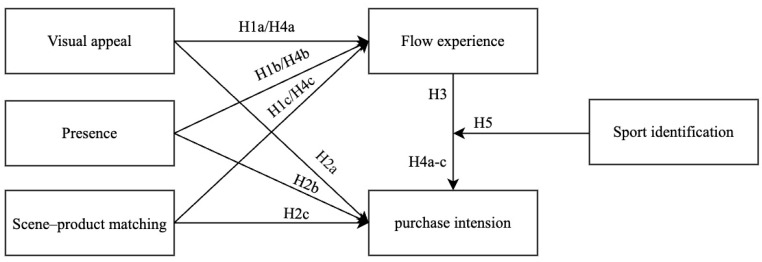
Research model of scene features influencing flow experience and purchase intentions.

Based on the above discussion, Figure 1 summarizes our research model. We examine the influence of scene features (i.e., visual appeal, presence, and scene–product matching) on consumers’ flow experience and purchase intention and the impact of flow experience on purchase intention. Moreover, we used sport identification as the moderating variable to examine the effect between flow experience and purchase intention.

## 4. Method

### 4.1. Measurement

The questionnaire includes six latent variables with 19 self-report items, which are shown in Appendix A, and some demographic characteristics (e.g., gender, age, educational background, monthly income, and the most watched platform). To ensure the accuracy and practicability of the questionnaire, all measurement items were from previous scales, with appropriate modifications to fit the context of sporting goods e-commerce live streaming. Because data collection was in China, we followed the Back-Translation method proposed by Brislin (1970). The English items were first translated into Chinese by one translator and then translated back into English by another translator. We compared the two versions to ensure that the meaning has no manifest differences. Moreover, we invited six consumers with experience in watching sporting goods e-commerce live streaming and two employees in the e-commerce live streaming industry to complete the questionnaire and judge whether there are unclear semantic expressions and understanding ambiguity in the questionnaire items. All items of VA (Loiacono et al., 2007), PR (Zhao et al., 2015), SM (Park & Lin, 2020), FE (Koufaris, 2002), SI (S. P. Hong & Rhee, 2016), and PI (Dodds et al., 1991) were taken on a Likert’s five-point scale (1 = “strongly disagree” to 5 = “totally agree”).

### 4.2. Data Collection

The data were collected via an online questionnaire. In order to ensure the diversity and effectiveness of the samples collected in this study, those who have the experience of watching sporting goods e-commerce live streaming were selected as the survey target. Therefore, a screening question was included. An invited message with a link to the questionnaire was sent to relatives, friends, classmates, etc., through social software, using a snowball sampling method to collect more samples. Moreover, the questionnaire was also distributed to several anchor’s fan groups of sporting goods e-commerce live streaming. The respondents were asked to recall their recent experience watching sporting goods e-commerce live streaming and completed the questionnaire based on this experience. The data collection period was in spring 2023, and a total of 340 valid responses were obtained.

## 5. Results and Analysis

### 5.1. Descriptive Statistics

The demographic information of respondents is summarized in Table 2. Of the 340 valid survey samples, 186 (54.7%) were female. In terms of age, all age groups are distributed. The largest number of people were 18–24 years old (31.2%), and the main watching crowd was the young and middle-aged group under 40 years old (66.1%). More than 75% of the respondents hold a bachelor’s degree or above. The monthly income was mainly in the group of CNY “5000–10,000” (USD 700–1400, 30.3%). Over half of the respondents said that the most often watched sporting goods e-commerce live streaming platform was Douyin (52.6%), followed by Taobao (25.6%), both of which are mainstream e-commerce live streaming platforms in China. In summary, the watching crowd of sporting goods e-commerce live streaming shows the characteristics of younger, well-educated, and higher income on the whole, which is consistent with the crowd portrait of the platform most often watched by users (Douyin e-commerce—sports and outdoor industry). Therefore, the participants in this study are highly representative.

### 5.2. Measurement Model

SPSS 26.0 was used to examine the reliability of the scale, and confirmatory factor analysis (CFA) by AMOS 26.0 was adapted to test the measurement model. The standardized factor loading, composite reliability (CR), average variation extraction value (AVE), and Cronbach’s α value of each variable and item in this study are shown in Table 3. The reliability (Cronbach’s α value of each variable >0.80) and composite reliability of the scale (CR value ranged from 0.860 to 0.896, >0.70 threshold (Bagozzi & Yi, 1988)) are good.

The measurement model fit indices as follows: χ^2^/df = 2.273 (<3), REMSEA = 0.061 (<0.08), RMR = 0.026 (<0.05), GFI = 0.915 (>0.90), NFI = 0.941 (>0.90), IFI = 0.966 (>0.90), TLI = 0.957 (>0.90), CFI = 0.966 (>0.90), which indicated that the CFA model of sporting goods e-commerce live streaming conducted by this study fit well. When CR > 0.70, AVE > 0.50, and standardized factor loading >0.70, it can be considered that the scale has good convergence validity (Fornell & Larcker, 1981). The square root of the AVE value of each variable is greater than the correlation coefficient between the variable and other variables (Table 4), indicating that the internal correlation of each variable is greater than the external correlation (Hair et al., 2009). Therefore, the scale has good discriminative validity.

### 5.3. Structural Model

**Figure 2:**
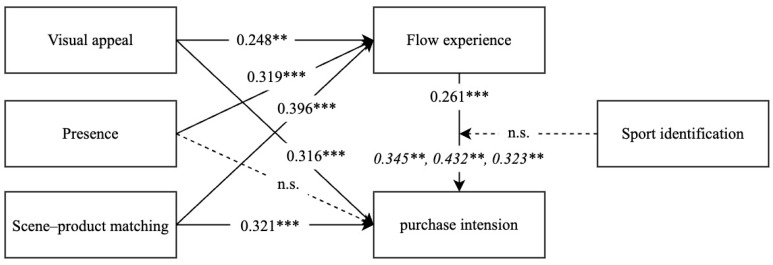
Results of the research model. Notes: *** *p* < 0.001, ** *p* < 0.01, n.s., no significant. The mediation effect (H4a–c) is shown in italics.

Structural equation modeling (SEM) was used to analyze the path influence relationship between the variables and test proposed hypotheses one by one in this study. The model fit indices for the structural model are as follows: χ^2^/df = 2.444 (<3), RMSEA = 0.065 (<0.08), RMR = 0.026 (<0.05), GFI = 0.924 (>0.9), NFI = 0.948 (>0.90), IFI = 0.969 (>0.90), TLI = 0.960 (>0.90), CFI = 0.968 (>0.90). Thus, the fit of the overall model is fairly good. The results of path coefficients, t-values, and significance of each hypothesis are shown in Table 5. From the perspective of the path that scene characteristics affect FE, VA (*β* = 0.248, *p* = 0.008), PR (*β* = 0.319, *p* < 0.001), and SM (*β* = 0.396, *p* < 0.001), H1a, H1b, and H1c are supported. From the perspective of the path that scene characteristics affect PI, VA (*β* = 0.316, *p* = 0.009), PR (*β* = −0.034, *p* = 0.751), and SM (*β* = 0.321, *p* = 0.003), H2a and H2c are supported, and H2b is refused. Moreover, the results show that FE is found to contribute positively to PI (*β* = 0.261, *p* = 0.036), and H3 is supported. In addition, the scene features of SM have the largest direct effect on FE and PI. The variances explained (R^2^) for FE and PI are 0.669 and 0.815, respectively. The values demonstrate that all of the factors in the research model can well explain the formation of the dependent variables. The results of the research model are shown in Figure 2.

### 5.4. Mediation Analysis

The bootstrapping approach is applied to further explore whether the FE plays a mediating role between scene features of sporting goods e-commerce live streaming (VA, PR, SM) and PI. We set the generation of 5000 bootstrap samples and used bootstrapping confidence intervals (CIs) including bias-corrected to 95% CI and percentile 95% CI to test total, direct, and indirect effects (Preacher & Hayes, 2004). The test results of the mediation effect are shown in Table 6. Firstly, FE has a significant mediating effect between VA and PI; 95% CI does not contain zero; the mediating effect accounts for 45.3%, while the direct effect accounts for 54.7%, which is a partial mediation. Secondly, FE has a significant mediating effect between PR and PI; but 95% CI of direct effect contains zero; the mediating effect accounts for 72%, while the direct effect accounts for 28%, which is a full mediation. Thirdly, FE has a significant mediating effect between SM and PI; 95% CI does not contain zero; the mediating effect accounts for 44.4%, while the direct effect accounts for 55.6%, which is a partial mediation. Therefore, the H4a, H4b, and H4c are supported.

### 5.5. Moderation Analysis

The hierarchical multiple regression is applied to examine whether the SI plays a moderating role between FE and PI. Firstly, the PI was set dependent variable, then gender, age, education, and income were set as control variables, which formed model 1. Secondly, the independent variable (FE) and moderating variable (SI) were added to model 1 to form model 2. Thirdly, the interaction term (FE × SI) was added to model 2 to form model 3. The test results of the moderation effect are shown in Table 7. The regression coefficient of the interaction term (FE × SI) is 0.001 (*t* = 0.16, *p* = 0.987), which means the interaction term has no significant influence on PI; moreover, the R^2^ of model 2 and model 3 have no significant change, indicating that the explanatory power of the model is weak. Therefore, the H5 is refused.

## 6. Discussion and Implications

### 6.1. Discussion of Findings

This study has developed a model of sporting goods e-commerce live streaming based on the S-O-R model and analyzed the impacts of scene features of visual appeal, presence, and scene–product matching on flow experience and purchase intention. In addition, this study further explored the mediating and moderating effects of the research model. A total of 11 hypotheses were proposed, among which H2b and H5 were refused, while the remaining hypotheses aligned with the analysis results.

A theory is a generalized explanation of how nature works. Kerlinger (1973) defined theory as “a set of interrelated constructs, concepts, definitions, and propositions that present a systematic view of phenomena by specifying relations among variables, with the purposes of explaining and predicting the phenomena” (p. 9). According to Mastromartino et al. (2024) and J. J. Zhang et al. (2015), there are two key threads theorizing to explain a phenomenon: (a) constructive definition of concepts and (b) examination of inter-concept relationships. In the current study, three dimensions (i.e., VA, PR, and SM) were first identified as relevant and representative of the concept of scene features; then, they were found to have a significant positive effect on FE (H1a–c). These findings are consistent with previous studies (e.g., J. Chen et al., 2009). The scene of sporting goods e-commerce live streaming is similar to authentic consume context, which lets the consumer into a state of concentration, further generating more product experience of visual and auditory. Moreover, comparing the path coefficient, SM tends to be more influential compared to VA and PR on FE. The higher the matching degree between the scene and sporting goods, the more predictive the presentation of sporting goods, which can decrease the cognitive load of consumers on sporting goods and immerse them in the real-use scene of sporting goods. These findings reinforce the importance of building congruence between scene and product features. Additionally, the variances explained (R^2^) for FE and PI are above 0.6. However, a high R^2^ value does not necessarily mean that the model has good predictive power in practical situations. This research did not introduce more relevant control variables (e.g., price sensitivity, brand familiarity) for robustness testing, which may have a significant impact on purchase intention, which is suggested for future studies.

The scene features of VA and SM have a significant positive effect on PI (H2a and H2c), which is consistent with previous studies (Shang et al., 2023; B. Zhang et al., 2021). In the process of live streaming shopping, most of the information perceived by consumers is visual information, and the scene of visual accessibility and attractiveness in sporting goods e-commerce live streaming can arouse consumers’ emotions, thereby improving consumers’ purchase intention. In addition, the match between the scene and the sporting goods will allow consumers to automatically substitute into the live context and generate the desire to own the sporting goods. However, the scene features of PR has no significant effect on PI (H2b), which echoes the conclusion of Zhao et al. (2015). The PR allows consumers to feel that they are shopping in a brick-and-mortar store to a certain extent, which is not enough to directly induce consumers’ purchase intention for sporting goods. The consumer is relatively rational and objective, even though they are in a live streaming shopping context similar to brick-and-mortar stores; they need more factors to stimulate the purchase intention for sporting goods. Moreover, when the scene of sporting goods e-commerce live streaming is deliberately created as a shopping context, consumers who want to watch e-commerce live streaming to get pleasure may have antipathy.

This study provides empirical evidence on the impact of FE on PI in sporting goods e-commerce live streaming (H3), which is consistent with the findings (Herrando et al., 2022; S. Huang et al., 2021). When consumers watch and immerse themselves in the sporting goods e-commerce live streaming, a strong flow experience will be generated, further improving consumers’ purchase intention. Moreover, the FE has a mediating role between the scene features of sporting goods e-commerce live streaming (VA, PR, and SM) and PI (H4a–c). These findings confirm that flow experience has a good predictive effect on consumers’ purchase intention in the online shopping context, including the newly emerging e-commerce live streaming in recent years. Furthermore, this study provides empirical evidence for the path relationship of “scene features of e-commerce live streaming—flow experience—purchase intention” from the field of sporting goods.

The SI has no significant moderating effect between FE and PI, contrary to our hypothesis. This finding may be related to the evolving landscape of sports consumption in contemporary society. With the advancement of national fitness programs, there has been a significant increase in public awareness and demand for sports. However, this heightened awareness does not necessarily translate into a strong identification with sports. Even in the absence of a high degree of sports identification, consumers may still purchase sports goods for various other motivations, such as health needs or social demands. The study reveals that the primary audience for e-commerce live streaming of sports goods is young people, whose purchasing motives may not be entirely linked to sports identification. Young consumers may purchase sports goods for reasons such as supporting domestic products, a preference for sports trends or fashion culture, or impulsive buying. These findings suggest that sports identification may not be the sole or even the most significant factor driving the purchase of sports goods among young consumers. Future research should explore whether consumer involvement, brand attachment, or social influence better moderates this relationship.

### 6.2. Theoretical and Practical Implications

Confirming the applicability of the S-O-R model in the sports e-commerce live streaming setting, the findings of this study identify dimensions of scene features of e-commerce live streaming and highlight the significance of developing scene–product congruence features when designing, operating, and promoting live streaming programs while enhancing immersive involvement. Specifically, these theoretical and practical contributions are discussed below.

Firstly, different product categories and settings of consumption will influence consumers’ purchase intention. However, previous studies mainly focused on clothing, cosmetics, fresh produce, and travel products, yet the research was focused on sporting goods, which expanded the scope of research in the field of e-commerce live streaming. Secondly, previous literature mainly focused on the characteristics of anchors, while ignoring the features of the scene. Joshua (1985) indicated that the scene constructed by media is a context that influences people’s social behavior. This study initially provides empirical evidence on the impact of scene features on consumers’ purchase intention. Thirdly, although flow theory has been studied for a long time and has been examined in different contexts, this study immersed flow experience in the S-O-R model and introduced it into the field of sporting goods. More specifically, this study identified the existence of the mediating role of flow experience in sporting goods e-commerce live streaming.

This research also generated several practical implications for sporting goods e-commerce live streaming, reminding us of some aspects that need attention. Firstly, an attractive live streaming scene is created. Visually attractive live streaming scenes can not only increase the probability of consumers entering the live streaming room but also increase the watching time of consumers staying in the live streaming room. The scene of sporting goods e-commerce live streaming should meet the needs of attracting consumers’ attention, so that users who browse on the live streaming platform but have no purchase plan can enter the e-commerce live streaming room and become potential consumers.

Secondly, it is constructive to build an immersive live streaming scene. The subject of sporting goods e-commerce livestreaming (e.g., brand, vendor, and anchor) can build the scene from the layout of the scene, close-up, music, lighting, and other aspects that strengthen the consumer perception of the authenticity of sporting goods. Moreover, brick-and-mortar stores as the scene of e-commerce live streaming are used to create an authentic shopping context and improve consumers’ recognition and trust. For example, sporting clothing e-commerce live streaming can use the fitting room as a scene to improve consumers’ presence and make up for the defect that unable to feel the physical products in an e-commerce live streaming context. In addition, the platforms (e.g., Douyin, Taobao) can apply more emerging technologies to the e-commerce live streaming rooms and bring consumers a richer, more diverse, and more vivid watching experience.

Thirdly, it is necessary to have scene–product matching to enhance consumers’ purchase intentions. The sporting goods e-commerce live streaming can use the authentic outdoor environment as a scene to improve consumers’ purchase intention. For example, the scene of Snow Mountain matches many outdoor products (e.g., hardshell jackets, down jackets, and softshell pants). Anchors can display functional characteristics such as wind and water resistance and scratch and wear resistance in a full range of products in the snow mountain scene, so that consumers have a deeper understanding of the product and improve consumers’ willingness to buy the product. A/B testing has investigated how background music placement (S. Zhang et al., 2023) and background visual complexity (Tong et al., 2022) influence consumers’ purchase intentions. Future research may draw on this methodological approach to examine the impact of scene–product matching.

### 6.3. Limitations and Future Directions

Although this study has made an exploration in the field of sporting goods e-commerce live streaming, it is not without limitations. Firstly, snowball sampling through personal networks to recruit participants may introduce selection bias. Future research should consider employing random sampling or stratified sampling to yield a more representative dataset. This research sample comprises mainly young to middle-aged consumers. The reality is that consumers of different ages and backgrounds may have different attitudes towards emerging consumption modes (Tellis et al., 2009); thus, in-depth segmentation studies are suggested for future investigations. Secondly, this study was only focused on Chinese consumers and China’s e-commerce live streaming platforms. However, other countries and regions such as the United States and Europe are taking comparatively smaller steps in the expansion of livestreaming commerce. Amazon and YouTube have been enhancing the capabilities of their websites and reviewing consumers’ reactions to e-commerce livestreaming. Future research can apply the theoretical model proposed in this study to other platforms (e.g., Twitter, Amazon, and TikTok) and other marketplaces (e.g., the United States, Europe, and other Asian regions) for comparative analyses. Thirdly, this research design employs the S-O-R model and focuses on basic scene features designed by the business (i.e., VA, PR, and SM), which omits interactive features of e-commerce live streaming of sporting goods. Moreover, the moderating role of sport identification was not found to be present in this research. Future studies should expand the scope of the study, explore other related variables, and even adopt additional theories (e.g., the Technology Acceptance Model and the Uses and Gratifications Theory) to seek for more comprehensive understanding of e-commerce consumer behavior. Finally, this study only utilized cross-sectional self-reported survey data to assess consumers’ flow experience and purchase intention. However, the actual behavior cannot be accurately measured. Future research can use a longitudinal study tracking consumer behavior (e.g., actual purchase records, dwell time analytics on live streams) or employ neuroscience techniques to make objective measurements, such as electroencephalograms, functional magnetic resonance, galvanic skin response sensors, and electrocardiograms (Herrando et al., 2022) to provide stronger causal inferences.

## Figures and Tables

**Table 1:** The dimensions of flow experience.

Number of Dimensions of the Flow Experience	Scholars	Characteristics
Single	Hoffman and Novak (1996)	Flow
Two	Ghani and Deshpande (1994)	Intense concentration, enjoyment
	Smith and Sivakumar (2004)	Intensity, duration
Three	Koufaris (2002)	Perceived control, enjoyment, concentration
Four	Webster et al. (1993)	Control, attention focus, curiosity, intrinsic interest
Five	Engeser (2012)	Merging of action and awareness, centering of attention, loss of self-consciousness, feeling of control in a task with coherent, noncontradictory demands, autotelic nature of the activity
Multiple	Czikszentmihalyi (1996)	Challenge–skills balance, action–awareness merging, clear goals, unambiguous feedback, concentration on the task at hand, sense of control, loss of self-consciousness, transformation of time, autotelic experience

**Table 2:** Demographics of respondents (*N* = 340).

Type	Item	Frequency	Percentage
Gender	Male	154	45.3%
	Female	186	54.7%
Age	18–24	106	31.2%
	25–30	56	16.5%
	31–40	64	18.8%
	41–50	67	19.7%
	Over 50	47	13.8%
Education background	Second school or below	15	4.4%
	High school	68	20.0%
	Bachelor	223	65.6%
	Master and PhD	34	10.0%
Monthly income (CNY)	Under 3000	99	29.1%
	3001–5000	98	28.8%
	5001–10,000	103	30.3%
	Over 10,001	40	11.8%
The most often watched platform	Taobao	87	25.6%
	Jingdong	34	10.1%
	Pinduoduo	16	4.7%
	Douyin	179	52.6%
	Kuaishou	14	4.1%
	Xiaohonghsu	10	2.9%

**Table 3:** Reliability and convergent validity.

Variable	Item	Std. Factor Loading	CR	AVE	Cronbach’s α
Visual appeal (VA)	VA1	0.752	0.861	0.675	0.858
	VA2	0.854			
	VA3	0.854			
Presence (PR)	PR1	0.858	0.890	0.730	0.888
	PR2	0.811			
	PR3	0.892			
Scene–product matching (SM)	SM1	0.865	0.896	0.741	0.896
	SM2	0.861			
	SM3	0.857			
Flow experience (FE)	FE1	0.813	0.896	0.683	0.890
	FE2	0.780			
	FE3	0.856			
	FE4	0.855			
Sport identification (SI)	SI1	0.764	0.881	0.713	0.878
	SI2	0.875			
	SI3	0.889			
Purchase intention (PI)	PI1	0.831	0.860	0.672	0.857
	PI2	0.837			
	PI3	0.790			

**Table 4:** Discriminant validity.

	VA	PR	SM	FE	SI	PI
VA	0.821					
PR	0.735 **	0.854				
SM	0.734 **	0.705 **	0.861			
FE	0.748 **	0.756 **	0.773 **	0.827		
SI	0.547 **	0.527 **	0.480 **	0.509 **	0.845	
PI	0.680 **	0.623 **	0.680 **	0.682 **	0.625 **	0.820

Notes: Diagonal elements are the square root of AVE; others are correlation coefficients. ** *p <* 0.01.

**Table 5:** Test results of direct effect.

Hypothesis	Path Relationship			Std. Coefficient	S.E.	t-Value	*p*	Results
H1a	VA	→	FE	0.248	0.093	2.673	0.008	Supported
H1b	PR	→	FE	0.319	0.071	3.897	***	Supported
H1c	SM	→	FE	0.396	0.075	5.172	***	Supported
H2a	VA	→	PI	0.316	0.117	2.623	0.009	Supported
H2b	PR	→	PI	−0.034	0.091	−0.318	0.751	Refused
H2c	SM	→	PI	0.321	0.102	2.968	0.003	Supported
H3	FE	→	PI	0.261	0.120	2.093	0.036	Supported

Notes: *** *p* < 0.001.

**Table 6:** Test results of mediation effect.

Effect Type	Path Relationship	Effect Size β	SE	Bias-Corrected 95% CI		Percentile 95% CI		Percentage
				Lower	Upper	Lower	Upper	
Total effect 1	VA→PI	0.763	0.087	0.602	0.946	0.598	0.944	—
Direct effects 1	VA→PI	0.418	0.156	0.134	0.765	0.128	0.756	54.7%
Indirect effects 1	VA→FE→PI	0.345	0.120	0.113	0.599	0.103	0.587	45.3%
Total effect 2	PR→PI	0.600	0.065	0.473	0.729	0.473	0.729	—
Direct effects 2	PR→PI	0.168	0.105	−0.022	0.397	−0.023	0.396	28.0%
Indirect effects 2	PR→FE→PI	0.432	0.091	0.264	0.629	0.252	0.614	72.0%
Total effect 3	SM→PI	0.728	0.750	0.587	0.879	0.588	0.879	—
Direct effects 3	SM→PI	0.405	0.141	0.158	0.716	0.153	0.708	55.6%
Indirect effects 3	SM→FE→PI	0.323	0.121	0.093	0.571	0.082	0.559	44.4%

Notes: VA, visual appeal; PR, presence; SM, scene–product matching; FE, flow experience; PI, purchase intention.

**Table 7:** Test results of moderation effect.

Variable		PI		
		M1	M2	M3
Control variable	Gender	−0.081	0.012	0.012
	Age	0.171 **	0.011	0.011
	Education	0.124 *	0.046	0.046
	Income	0.006	−0.030	−0.030
Independent variable	FE		0.499 ***	0.499 ***
Moderating variable	SI		0.370 ***	0.370 ***
Interaction term	FE × SI			0.001
	R^2^	0.036	0.572	0.572
	Adj. R^2^	0.024	0.564	0.563
	F	3.115 *	74.081 ***	63.308 ***

Notes: All data are standardization coefficient beta. *** *p* < 0.001, ** *p* < 0.01, * *p* < 0.05.

## Data Availability

The original contributions presented in the study are included in the article, further inquiries can be directed to the corresponding author.

## Appendix A. Constructs and Measurement Items

**Construction**	**Code**	**Items**	**Reference**
Visual appeal (VA)	VA1	Scene of sporting goods e-commerce live streaming is visually pleasing	(Loiacono et al., 2007)
	VA2	Scene of sporting goods e-commerce live streaming design visually pleasing design	
	VA3	Scene of sporting goods e-commerce live streaming is visually appealing	
Presence (PR)	PR1	I felt the sporting goods in front of me	(Zhao et al., 2015)
	PR2	I felt I was surrounded by the scene of e-commerce live streaming	
	PR3	The scene of e-commerce live streaming gave me a feeling of being here	
Scene–product matching (SM)	SM1	The scene of live streaming matches well with sporting goods	(Park & Lin, 2020)
	SM2	The matching of the scene of live streaming with sporting goods is natural	
	SM3	The scene of live streaming is highly appropriate for sporting goods	
Flow experience (FE)	FE1	I felt time went fast when watching e-commerce live streaming	(Koufaris, 2002) (H. Chen, 2006)
	FE2	I was deeply engrossed in the e-commerce live streaming and ignore other things	
	FE3	I felt enjoyment when watching e-commerce live streaming	
	FE4	I felt interested when watching e-commerce live streaming	
Sport identification (SI)	SI1	It is important to me to be a part of my favorite sport	(S. P. Hong & Rhee, 2016)
	SI2	My friends view me as a strong fan of my favorite sport	
	SI3	I see myself as a strong fan of my favorite sport	
Purchase intention (PI)	PI1	I have a high probability of considering buying sporting goods recommended by e-commerce live streaming	(Dodds et al., 1991)
	PI2	I am willing to buy sporting goods recommended by e-commerce live streaming	
	PI3	I would like to recommend the sporting goods in e-commerce live streaming to others	
